# Microbial diversity and water quality changes in mangrove sediments in Quanzhou Bay

**DOI:** 10.3389/fmicb.2026.1743704

**Published:** 2026-02-10

**Authors:** Wenzhou Zhang, Huixuan Jiang, Qingyu Zhu, Ziying Shi, Wenbiao Chen, Xiaowen Xu, Fei Peng, Yulang Chi

**Affiliations:** 1 School of Pharmacy, Quanzhou Medical College, Quanzhou, China; 2 College of Oceanology and Food Science, Quanzhou Normal University, Quanzhou, China; 3 Department of Basic Medicine, Quanzhou Medical College, Quanzhou, China

**Keywords:** environmental drivers, mangrove sediments, microbial diversity, Quanzhou Bay, spatial variation

## Abstract

This study investigated the diversity, composition, and environmental drivers of bacterial communities in the mangrove sediments of Quanzhou Bay, a subtropical estuary under anthropogenic pressure. Using high-throughput sequencing of the 16S rRNA gene, we analyzed samples from four sites (Fengze-FZ, Jinjiang-JJ, Luojiang-LJ, and Shishi-SS) representing a gradient of terrestrial influence and environmental conditions. The bacterial communities were predominantly composed of Pseudomonadota and Chloroflexi, a pattern consistent with global mangrove ecosystems but with distinct local structuring. Beta-diversity analyses (NMDS/PCA) revealed a significant spatial divergence, with the FZ site forming a distinct cluster separate from JJ, LJ, and SS, correlating with its unique environmental profile. Redundancy analysis (RDA) identified dissolved oxygen (LDO) and salinity as the key environmental factors shaping community structure. Functional prediction indicated a conserved potential for core metabolic processes (e.g., amino acid biosynthesis, bacterial chemotaxis) across sites, suggesting functional redundancy, while differences in the relative abundance of these pathways pointed to adaptive metabolic adjustments along the environmental gradient. Our findings demonstrate that the sedimentary microbial community structure in Quanzhou Bay is primarily shaped by localized environmental heterogeneity, providing critical insights into the microbial ecology of mangroves in urbanized coasts and a baseline for assessing ecosystem health and biogeochemical functioning under anthropogenic influence.

## 1 Introduction

The mangrove forest in Quanzhou Bay is located in Quanzhou, Fujian Province, with geographical coordinates of 118°37′45″ ~ 118°42′44″ east longitude and 24°47′37″ ~ 24°57′29″ north latitude. As one of the important bays in Fujian Province, Quanzhou Bay has become an important area for ecosystem research with its unique geographical location and rich natural resources. In recent years, with the rapid development of economy, the process of industrialization and urbanization around Quanzhou Bay has been accelerated, and the ecological environment has been affected to varying degrees. In this context, mangroves, as an important wetland ecosystem, not only make an important contribution to marine biodiversity, but also play an indispensable role in water purification, carbon storage and coastal protection (Cheung et al., 2018). The health of mangrove ecosystem is closely related to the microbial diversity in its sediments (Bhattacharyya et al., 2015). As the most basic life form in the ecosystem, microorganisms bear many ecological functions such as nutrient circulation and energy transformation. They not only play a key role in the process of organic matter decomposition and nitrogen transformation, but also have sensitive reactions to environmental changes (Shu and Huang, 2022). Therefore, studying the microbial diversity of mangrove sediments in Quanzhou Bay can help us understand the health status of this ecosystem and its adaptability to external environmental changes. This understanding is informed by studies across Southeast Asia and China, which have established that mangrove sediment communities are typically dominated by phyla such as Pseudomonadota, Bacteroidota, and Chloroflexi, and are highly responsive to environmental gradients including salinity, nutrients, and pollutants (He et al., 2025; Zhang et al., 2023).

However, microbial responses to combined, location-specific stressors-particularly the mixed-pressure gradients from non-point source pollution in urbanizing coasts-remain less predictable and require targeted study. Quanzhou Bay presents a distinctive case in this context. Unlike heavily industrialized estuaries or pristine reserves, the mangroves along its inner shores, fed by the Luoyang and Jinjiang Rivers, are subject to a distinct gradient of pollution dominated by domestic and agricultural sewage. This setting, combined with variations in local hydrodynamics and vegetation, creates a unique natural experiment. Most prior regional studies have focused on single stressor types or broader surveys. Therefore, our work is designed to systematically examine how sedimentary bacterial communities assemble across multiple sites within Quanzhou Bay representing different positions along this defined pollution-vegetation gradient. By integrating high-throughput sequencing with in-situ physicochemical analysis, we aim to (1) characterize the site-specific bacterial community structure, (2) identify the key environmental drivers, and (3) evaluate the ecological implications. This provides a critical assessment of Quanzhou Bay while contributing a relevant case study on microbial adaptation to pervasive, non-industrial anthropogenic stress in subtropical mangroves.

With the progress of various research techniques, in addition to the traditional isolation and culture techniques, metagenome and metatranscriptome methods are widely used in the analysis of microbial communities (Kannan et al., 2023; Priya et al., 2021). It has been found that less than 5% of the microbial communities in mangroves are described, and usually less than 1% are culturable microorganisms (Dhariwal et al., 2017). Mangrove ecosystem is very important. It participates in the regulation of carbon cycle, nitrogen cycle, phosphorus cycle, sulfur cycle and other processes, and is an important place to discover new natural products, functional genes and carry out microbial investigation (Liu et al., 2019; Nimnoi and Pongsilp, 2022). However, the microbial ecology in mangrove sediments is constantly affected by environmental factors such as polluted water. Although mangroves have strong ecological restoration ability, sewage, organic pollutants, microplastics and trace metals still greatly affect the microbial community, change the diversity of microorganisms, and even affect the population of certain microorganisms (Szafranski and Granek, 2023). Recent studies show that the structure and function of microbial community in mangrove sediments are affected by many factors, including water quality changes, sediment properties and human activities (Li et al., 2011).

In this context, soil sediments from four mangrove areas along the pollution gradient in Quanzhou Bay were collected from four mangrove areas along Quanzhou Bay were collected, and the relationship between bacterial communities and environmental factors was studied. The purpose of this paper is to explore the characteristics of bacterial community diversity in soil sediments in the mangrove areas along the Luoyang River and Jinjiang River in Quanzhou Bay, which have different vegetation and domestic sewage pollution levels, and to evaluate the advantages and abundance changes of microbial communities in different locations. Through the study of this topic, we hope to reveal the health status of mangrove ecosystem in Quanzhou Bay, clarify the important role of microorganisms in the ecosystem, and provide scientific support for future ecological restoration and protection. With the increasing influence of global climate change and human activities, a deep understanding of the relationship between mangroves and their microbial communities is not only of great significance to the ecological protection in Quanzhou Bay, but also provides valuable experience and reference for the study of mangrove ecosystems around the world.

## 2 Materials and methods

### 2.1 Collection of samples

**Figure 1:**
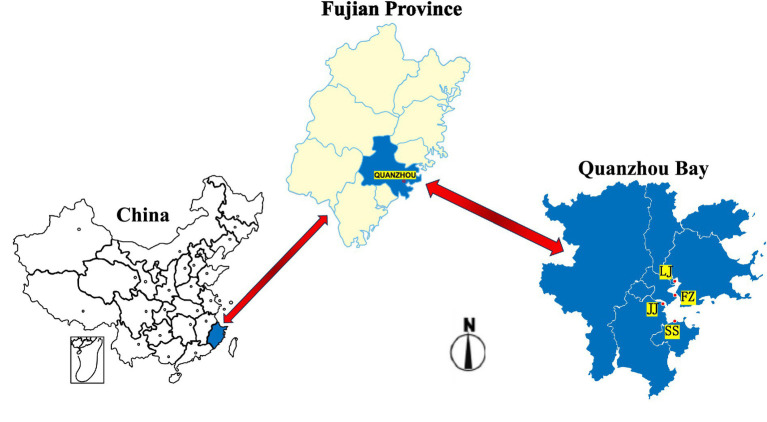
Sampling sites of mangrove sediments in Quanzhou Bay.

The sampling was conducted on August 13, 2024, in the Quanzhou Bay. To capture key spatial and environmental gradients within the bay, samples were strategically collected from four designated areas: Fengze District (FZ), Jinjiang City (JJ), Luojiang District (LJ), and Shishi City (SS), as illustrated in Figure 1 and Table 1. These sites were selected to represent a combination of factors: (i) proximity to the two major freshwater inputs (FZ near the Luoyang River estuary; JJ near the Jinjiang River estuary), and (ii) position along the inner-outer bay gradient (LJ in an interior area; SS in the outer bay). This design aims to encompass a range of potential influences from riverine input, terrestrial runoff, and bay hydrodynamics. The mangroves selected in this study are mainly artificial forests, and the main plant species are *Paulownia* and *Kandelia candel*. The depth of sample collection is about 10 cm, and the amount of sediments collected for each sample is about 0.5 kg. After thorough mixing, the samples are immediately stored in an ice box. After arriving at the laboratory, about 50 g of the mixed sample was put in a 50 mL sterile centrifuge tube for soil DNA extraction, and the rest of the soil was kept in a sterile polyethylene bag for soil physical and chemical properties analysis, and the rest was immediately stored in a refrigerator at −80 °C (Zhang et al., 2015). For the collection of interstitial water, 200 mL of pore water is collected at each station, and some physical and chemical parameters are determined immediately. Another 200 mL is stored in an ice box and returned to the laboratory to determine the physical and chemical parameters (Ding et al., 2024).

**Table 1:** Latitude and longitude of the sampling points.

Sampling points	Longitude	Latitude
FZ	118°40′40”	24°54′15”
JJ	118°38′52”	24°51′50”
LJ	118°40′56”	24°57′35”
SS	118°39′27”	24°48′26”

### 2.2 Determination of physical and chemical indexes

The parameters of pH, CDC, LDO and salinity of the water sample pass the portable measuring instrument (Hach Company, Loveland, CO, USA). NH_4_-N(Ammoniacal nitrogen)and NO_3_-N(Nitrate nitrogen)were determined by standard methods (Xu et al., 2023).

### 2.3 Sample DNA extraction, PCR amplification and 16SrDNA assay

Acquisition of PCR products: Add 15 μL Phusion High-Fidelity PCR Master Mix (New England Biolabs), 0.2 μM primer and 10 ng genomic DNA template to all PCR mixed solutions, and perform the first denaturation at 98 °C for 1 min. Then 30 cycles were carried out at 98 °C (10 s), 50 °C (30 s) and 72 °C (30 s), and finally kept at 72 °C for 5 min. The V4 variable region of 16S rRNA gene was amplified by PCR using primers 515F (GTGCCAGCMGCCGCGGTAA) and 806R (GGACTACHVGGGTWTCTAAT) (Nimnoi and Pongsilp, 2022). Library construction and computer sequencing: use TRUSEQ DNA PCR-FREE SAMPLE PREPARATION Kit to build the library. After the constructed library is quantified by Qubit and Q-PCR, use NovaSeq 6000 to perform computer sequencing on PE 250.

### 2.4 Bioinformatics analysis

Bioinformatic processing was performed using a QIIME2-based analysis pipeline (version 2019.1). Raw paired-end reads were imported into QIIME2, and primer sequences were trimmed. Sequences were then quality-filtered, denoised, merged, and checked for chimeras using the DADA2 plugin within QIIME2 (Callahan et al., 2016), which directly produces high-resolution amplicon sequence variants (ASVs). To ensure direct comparability with a wide range of published studies on mangrove sediment microbiota, the resulting ASVs were subsequently clustered into Operational Taxonomic Units (OTUs) at a 97% similarity threshold using the vsearch plugin within QIIME2 (Caporaso et al., 2010; Li et al., 2022). This OTU-based approach was chosen because it aligns with the conventional reporting standard in the field, thereby enabling meaningful cross-study comparison of community diversity, structure, and environmental correlates—which are central to the ecological questions addressed here. A representative sequence from each OTU was selected for taxonomic classification. Taxonomic assignment was performed using the SILVA database (release 138.1) via the RDP classifier implemented in QIIME2, with a confidence threshold of 0.7. Following initial taxonomic assignment, the nomenclature for prokaryotic phyla and classes was updated to reflect current standards (e.g., “Proteobacteria” to “Pseudomonadota”). This update was performed through manual curation against the List of Prokaryotes with Standing in Nomenclature (LPSN) and the SILVA browser, not by re-analyzing sequences with a newer database version. Alpha-diversity indices (Shannon, Chao1, observed features, Faith’s PD and Simpson) were calculated based on the OTU table using R (version 4.3.1). Beta-diversity was assessed based on the Bray–Curtis dissimilarity matrix calculated from the OTU abundance table using the Vegan package in R (Lou et al., 2018). Non-metric multidimensional scaling (NMDS) and principal coordinate analysis (PCoA) based on unweighted UniFrac distance were performed to visualize community dissimilarities. The functional potential of the bacterial community was predicted from the OTU representative sequences using PICRUSt2 with reference to the KEGG Orthology (KO) database (Xie et al., 2021).

## 3 Results

### 3.1 Effects of mangrove sediments on microorganisms in different areas of Quanzhou Bay

**Figure 2:**
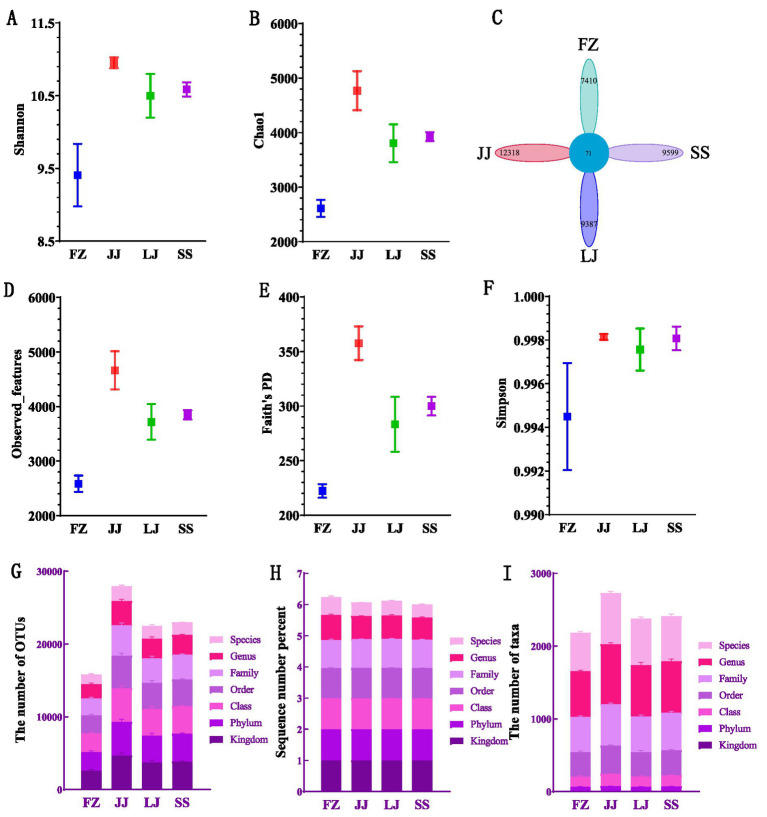
Alpha diversity indices and taxonomic annotation summary of sediment microbial communities. **(A–F)** Box plots of alpha diversity indices across four sampling sites (FZ, JJ, LJ, SS): **(A)** Shannon index, **(B)** Chao1 index, **(C)** Petal diagram displaying unique and shared OTUs among sites, **(D)** Observed features, **(E)** Faith’s phylogenetic diversity (PD), and **(F)** Simpson index. **(G–I)** Bar charts summarizing the taxonomic annotation efficacy: **(G)** The number of OTUs assigned at each taxonomic rank (from phylum to species), indicating how many OTUs could be classified at each level of resolution. **(H)** The percentage of total sequences that were successfully assigned at each taxonomic rank, reflecting the annotation coverage. **(I)** The number of distinct taxa (unique phyla, classes, orders, etc.) identified at each taxonomic rank.

Based on the analysis of diversity indices, including the Shannon, Chao1, observed features, Faith’s PD and Simpson (Figures 2A–F), in mangrove sediments from different areas of Quanzhou Bay, several key patterns were identified. Overall, the numerical trends of these indices across samples were largely consistent, suggesting similar levels of species richness among the sampled sites. Specifically, the JJ sample exhibited a high Shannon index, reflecting not only high species richness but also relatively even species distribution. Similarly, the Faith index, which incorporates phylogenetic distances to assess phylogenetic diversity, was also elevated in the JJ sample, indicating greater evolutionary divergence among taxa. In contrast, the Simpson index values exceeded 0.990 across all samples (Figure 2F), pointing to a community structure dominated by a few highly abundant species, with the remainder being relatively rare or less prevalent. Further analysis shows that after calculating the unique and common characteristics of each sample group according to the given abundance table, the number of operational taxonomic groups (OTU) shared by the data set is 71, while the number of OTU unique to JJ is the largest, with 12,318. The number of OTU unique to FZ is the least, which is 7,410 (Figure 2C). At different levels of species classification, the number of OTU (Figure 2G) and the number of taxa (Figure 2I) show the basic information of species annotation statistics, indicating that JJ’s species annotation is more comprehensive. In addition, the percentage of serial number (Figure 2H) shows that there is little difference among species in each sequence, which further verifies the consistency of species annotation. These results not only reflect the diversity of microbial communities in mangrove sediments in Quanzhou Bay, but also provide an important basis for understanding the uniqueness and commonness of microbial communities in different regions.

**Figure 3:**
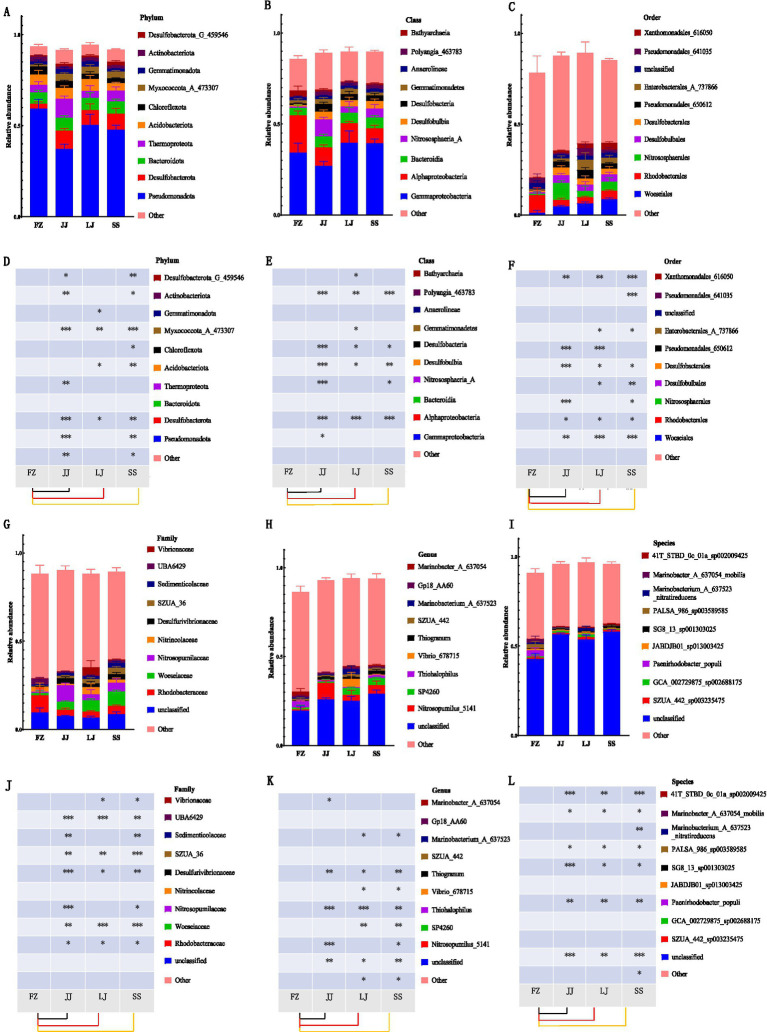
Sediment microbiota composition at different taxonomic ranks which are **(A,D)** phylum, **(B,E)** class, **(C,F)** order, **(G,J)** family, **(H,K)** genus, **(I,L)** species. *p < 0.05, **p < 0.01, ***p < 0.001.

This study further analyzed the composition of microbial communities in mangrove sediments in different areas of Quanzhou Bay, and revealed their significant differences at the level of phylum, class, order, family, genus and species (as shown in Figure 3; the complete relative abundance data for all identified taxa at each taxonomic level are provided in Supplementary Table S1; we focused on the top 10 taxa). At the phylum level (Figures 3A,D), the main microbial communities include Pseudomonadota (predominant), Desulfobacterota, Bacteroidota, Thermoproteota, and Acidobacteriota. Among them, Pseudomonadota has the highest content in the FZ region and the lowest in the JJ region; Desulfobacterota_I has the highest content in the JJ region and the lowest in the FZ region; Bacteroidota shows significant differences among the four regions; Thermoproteota is significantly higher in the JJ region than in other regions; Acidobacteriota has the highest content in the LJ region and the lowest in the SS region. At the class level (Figures 3B,E), the main groups are Gammaproteobacteria, Alphaproteobacteria, Bacteroidia, Nitrososphaeria_A, and Desulfobulbia, among which Bacteroidia shows no significant differences among different regions. At the order level (Figures 3C,F), the main groups include Woeseiales, Rhodobacterales, Nitrososphaerales, Desulfobulbales, and Desulfobacterales. Among them, only unclassified and Kiloniellales show no significant differences in all sediment samples. At the family level (Figures 3G,J), the main groups are Rhodobacteraceae, Woeseiaceae, Nitrosopumilaceae, Nitrincolaceae, and Desulfurivibrionaceae. Among them, Nitrosopumilaceae, UBA6911, GCA_2729495, and UBA5794 show no significant differences in all sediment samples. At the genus level (Figures 3H,K), the main groups include Nitrosopumilus_5141, SP4260, Thiohalophilus, Vibrio_678715, and Thiogranum. Among them, only Marinobacterium_A_637622, JABDJB01, and SZUA_442 show no significant differences in all sediment samples. At the species level (Figures 3I,L), the main groups are SZUA_442_sp003235475, GCA_002729875_sp002688175, Paenirhodobacter_populi, JABDJBO1_Sp013003425, and SG8_13_sp001303025. Among them, Paenirhodobacter_populi is significantly higher than other sediment samples.

**Figure 4:**
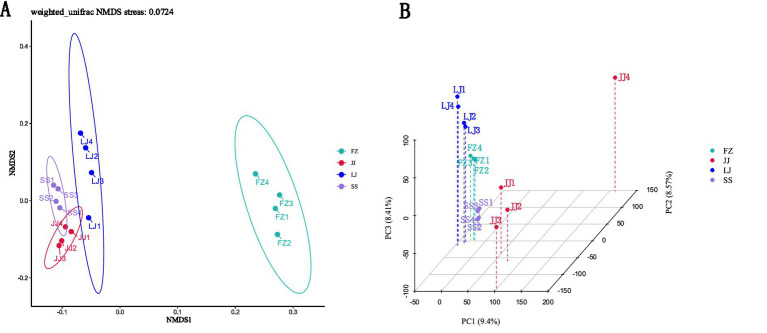
Environmental microorganism beta diversity analyses: **(A)** Non-metric multidimensional scaling (NMDS) and **(B)** principal component analysis (PCA).

**Figure 5:**
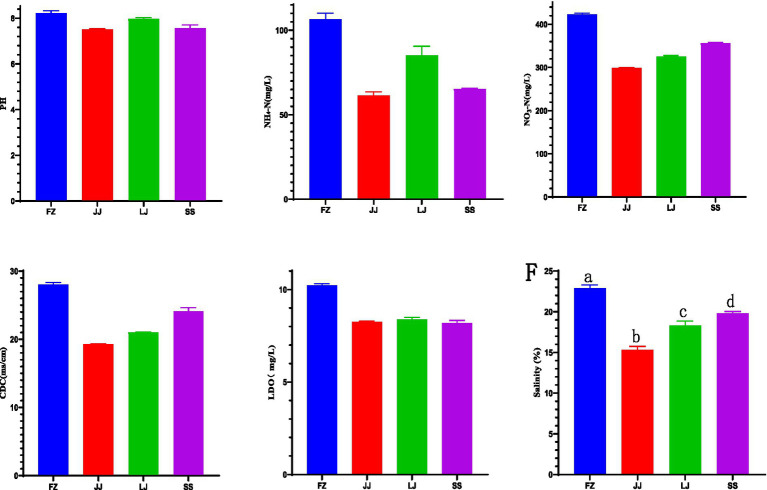
Different water quality factors include **(A)** pH, **(B)** NH_4_-N, **(C)** NO_3_–N, **(D)** CDC (conductivity), **(E)** LDO (dissolved oxygen), **(F)** salinity.

In addition, non-metric multidimensional scaling (NMDS) and principal component analysis (PCA) were used to analyze the beta diversity of microbial communities in the sediments. The NMDS analysis, based on Bray-Curtis dissimilarities, revealed a clear separation in community structure (Figure 4A). Samples from the SS, JJ, and LJ sites clustered together on the left side of the NMDS1 axis, indicating a higher degree of similarity among their microbial assemblages. In contrast, samples from the FZ site formed a distinct cluster on the right side of the NMDS1 axis, signifying a significantly different community composition. The PCA, a variance-based linear ordination method, provided a complementary perspective (Figure 4B). While also showing separation, the three-dimensional PCA plot resolved the samples from the LJ, FZ, and JJ sites into more distinct spatial positions. This divergence in results between NMDS and PCA is methodologically expected and informative. NMDS optimally represents the rank-order dissimilarities between samples, effectively highlighting the major pattern of FZ’s distinctness. PCA, by contrast, prioritizes axes that capture the maximum variance in the species abundance data, which may make it more sensitive to finer-scale variations driven by highly abundant or variable taxa, even among sites that share a broadly similar community structure (as seen in the NMDS clustering of SS, JJ, and LJ). Together, these analyses confirm the significant uniqueness of the FZ microbial community while also suggesting the presence of subtler, variance-driven gradients among the other sites. These patterns provide a crucial foundation for subsequent analysis linking community composition to specific environmental drivers in Quanzhou Bay. The distinct separation of the JJ sample along the PC2 axis (Figure 4B) aligns with its specific environmental context and community profile. This site exhibited the lowest concentrations of inorganic nitrogen (NH_4_ -N and NO_3_ –N, Figures 5B,C) among all samples. Correspondingly, its microbial community was characterized by a notably higher relative abundance of taxa within the phylum Thermoproteota (Figures 3A,D). This confluence of environmental and biological factors likely underpins its unique positioning in the ordination space.

### 3.2 Analysis of characteristic microbial taxa in mangrove sediments from different areas of Quanzhou Bay

**Figure 6:**
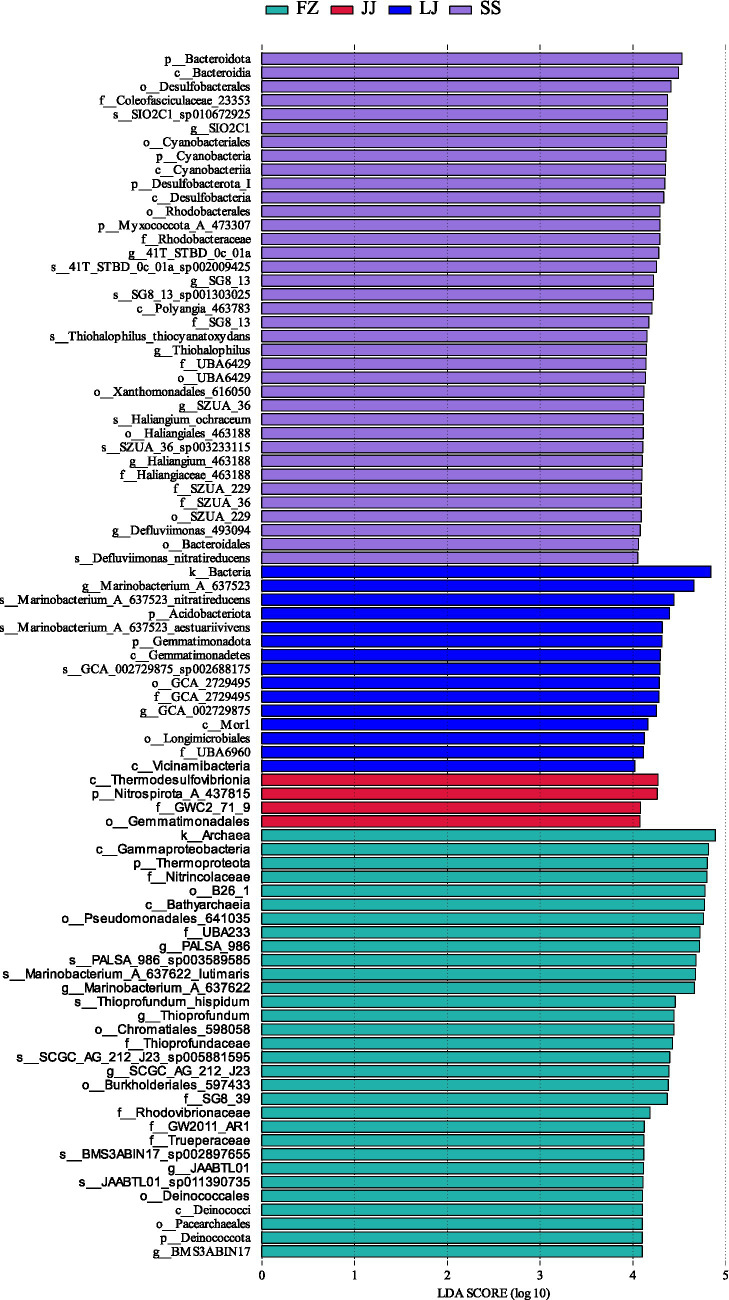
Analysis of characteristic taxa (covering taxonomic levels from kingdom to species) in different sites.

The analysis results of characteristic microbial taxa (covering taxonomic levels from kingdom to species) in different sediments are shown in Figure 6. Note: The characteristic taxa in Figure 6 involve multiple taxonomic levels (from kingdom to species). This is because the taxonomic identification accuracy of different microbial groups is limited by the completeness of reference databases: some groups can only be identified to higher levels (e.g., kingdom, phylum, class) due to insufficient reference information, while others can be resolved to the species level. We presented each taxon at its achievable highest taxonomic resolution to comprehensively display all significantly differentiated characteristic microbial groups.

The research findings indicate that there are a total of 87 significant microbial taxa in the mangroves of different regions in Quanzhou Bay. Among them, there are 37 characteristic taxa (predominantly identified to the species level) showing significant differences in the SS region, and the most significantly differentiated taxon (phylum level) is Bacteroidota. There are only four characteristic taxa in the JJ region, including *Thermodesulfovibrionia* (class level), *Nitrospirota_A_437815* (phylum level), *GWC2_71_9* (family level) and *Gemmatimonadales* (order level). The LJ region contains 15 characteristic taxa, with the main taxa including Bacteria (kingdom level), *Garinobacterium_A_637523* (species level), and *Marinobacterium_A_637523_nitratireducens* (species level). The number of characteristic taxa in the FZ region is 31, including Archaea (kingdom level), *Gammaproteobacteria* (class level), *Thermoproteota* (phylum level), etc.

### 3.3 Functional prediction of microbial communities in mangrove sediments in different regions of Quanzhou Bay

**Figure 7:**
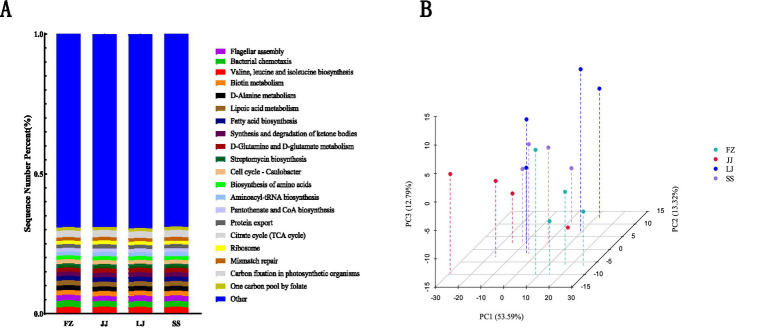
**(A)** Top 20 characteristic KEGG pathway profiles. **(B)** Principal component analysis (PCA).

The predicted functional profiles of the sedimentary microbial communities, based on the KEGG database, provide insights into their potential ecological roles within the mangrove ecosystem (Figure 7A). The complete KEGG functional pathway dataset corresponding to this figure has been provided in the Supplementary Table S2. Functions related to motility and environmental sensing, such as “Flagellar assembly” and “Bacterial chemotaxis,” were highly represented. This suggests an adaptive strategy for microorganisms to navigate the heterogeneous and dynamic sediment matrix, potentially optimizing their access to nutrients, electron acceptors, or favorable micro-niches, thereby influencing the spatial efficiency of organic matter decomposition and biogeochemical transformations. The most abundant predicted pathway was “Valine, leucine, and isoleucine biosynthesis.” The high potential for synthesis of these essential amino acids may indicate an active community involved in nitrogen assimilation and the processing of nitrogen-containing organic compounds, linking this functional trait directly to nitrogen cycling and the degradation of mangrove-derived detritus.

Principal Component Analysis (PCA) of the KEGG pathway profiles revealed significant variation in functional potential across sampling sites (Figure 7B). The first principal component (PC1, 53.59% variance) effectively separated the samples, with the FZ community displaying a notably distinct functional profile from the JJ, LJ, and SS communities along this axis. This functional divergence aligns with the distinct microbial composition observed at FZ (Figure 4) and correlates with its unique environmental setting. The second component (PC2, 13.32% variance) further captured variability, particularly within the JJ, LJ, and SS groups, indicating subtle functional differences even among sites with more similar community structures. These inter- and intra-group functional differences likely reflect adaptations to localized environmental conditions. For example, variations in the relative abundance of pathways related to osmotic stress response, specific nutrient transport systems, or pollutant degradation could be driven by site-specific gradients in salinity, nutrient concentrations, or types of anthropogenic input. This linkage between predicted function and environmental measurement underscores how localized pressures may shape the metabolic capabilities of sediment microbial communities, with implications for ecosystem-level processes like nutrient retention, carbon sequestration, and pollutant detoxification in Quanzhou Bay.

In summary, the predicted functional profiles of sedimentary microbial communities revealed a consistent core of metabolic capabilities (e.g., amino acid biosynthesis, bacterial chemotaxis) across all sites, yet exhibited clear site-specific patterns that correlated strongly with local environmental gradients. These trends, along with the primary environmental drivers identified through redundancy analysis, are synthesized in Table 2, providing an integrated overview of the structure–function-environment relationships in Quanzhou Bay mangrove sediments.

**Table 2:** Summary of site-specific microbial functional profiles and their primary environmental correlates in Quanzhou Bay mangrove sediments.

Site (code)	Location context	Distinctiveness in functional profile (KEGG pathways)	Key environmental correlates
FZ	Near Luoyang River estuary; minimal direct human activity.	Most distinct. Clearly separated from other sites along PC1 (53.59% variance, Figure 7B). Shares core pathways (e.g., amino acid biosynthesis) but with divergent abundance.	Positive correlation with all factors, especially LDO and salinity (Figure 8B).
JJ	Near Jinjiang River estuary; mixed freshwater–seawater zone.	Similar core pathways (Figure 7A) to LJ & SS, but shows subtle intra-group variability (PC2, 12.79% variance).	Negative correlation with measured environmental factors.
LJ	Interior bay area; low anthropogenic influence.	Similar core pathways (Figure 7A) to JJ & SS. Clusters with them in functional PCA (Figure 7B).	Negative correlation with measured environmental factors.
SS	Outer bay; strong marine influence.	Similar core pathways (Figure 7A) to JJ & LJ. Clusters with them in functional PCA (Figure 7B).	Negative correlation with measured environmental factors.
Overall trend	Gradient from riverine (FZ) to marine (SS) influence.	Functional redundancy in core metabolism across all sites; Adaptive divergence in functional structure driven by localized conditions.	LDO and salinity are the strongest drivers shaping both community structure and functional potential (Figure 8B, RDA).

### 3.4 Relationship between mangroves and water quality in different areas of Quanzhou Bay

The analysis of water quality changes in Quanzhou Bay coastal waters shows that the pH values of different mangrove areas are different, and the pH values of JJ and SS are not significant, ranging from 7.52 to 8.18 (Figure 5A). There are significant differences in NH_4_-N in sediments from different regions, among which the content of FZ is the highest, which is 105.50 mg/L, while the content of JJ is the lowest, which is 62.73 mg/L (Figure 5B). The content of NO_3_-N ranged from 299.00 mg/L to 422.75 mg/L, and there were significant differences among different regions, with the highest content of FZ and the lowest content of JJ (Figure 5C). The value of CDC in different places is 19.34 to 28.15 ms/cm, and there are significant differences in sediments in different areas (Figure 5D). In terms of LDO value, FZ is significantly different from other regions, but there is no significant difference between JJ, LJ and SS, and the content ranges from 8.15 to 10.23 mg/L (Figure 5E). Salinity also has obvious differences in different regions, with the content ranging from 15.13% to 23.00% (Figure 5F).

### 3.5 Relationship between microorganisms and water quality

**Figure 8:**
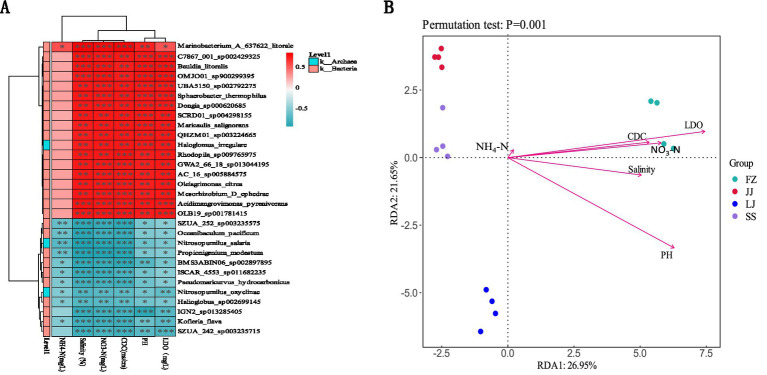
The relationship between sediment microbiota (at the genus level) and environmental factors. *R*-values (rank correlation) and *p*-values are obtained by calculation. *R*-values are shown in different colors in the figure, and if the *p*-value is less than 0.05, it is marked with *. The legend on the right is the color interval of different *R*-values. Meanwhile, the color bar on the left indicates the phylum classification to which the species belongs. *p < 0.05, **p < 0.01, ***p < 0.001. **(A)** Heat map of correlation between the environmental factors and generic level microorganisms, **(B)** RDA (redundancy analysis).

In order to evaluate the relationship between environmental factors and microbial community abundance, correlation heat map analysis and RDA (redundancy analysis) were carried out in this study, and the results are shown in Figure 8. The analysis shows that there are close correlations between 30 species of sediment microorganisms and 6 environmental factors. Among them, 18 kinds of sediment microorganisms are positively correlated with these environmental factors, while the other 12 kinds of sediment microorganisms are negatively correlated with them (Figure 8A). According to the results of RDA analysis, the explanation rates of community structure differences on the two ordination axes are 26.95% and 21.65%, respectively. Microbial communities have the highest correlation with LDO and the lowest correlation with NH_4_-N. In the FZ sample, the microbial community is positively correlated with six environmental factors, while the microbial communities in other samples are negatively correlated with these environmental factors (Figure 8B).

## 4 Discussion

As an important part of marine ecosystem, mangroves are mainly distributed in coastal areas, and their ecosystem service functions are widely recognized (Palit et al., 2022). Microbial communities in mangrove sediments play a key role in maintaining ecosystem functions (Dabala et al., 2023). Mangroves in Quanzhou Bay are mostly planted artificially, which is an important measure for ecosystem restoration. Previous studies have shown that the functional diversity of mangrove soil microorganisms will change with time, and its community structure is closely related to soil physical and chemical characteristics (Kamal et al., 2022; Li et al., 2022). However, the current research mostly focuses on the effects of soil physical and chemical properties, environmental factors and seasonal changes on the microorganisms in mangrove sediments, while the research on the diversity and distribution characteristics of microorganisms in mangrove sediments along Quanzhou Bay is relatively rare (Yang et al., 2023; Zhou et al., 2017).

In view of the special environment of mangrove area in Quanzhou Bay, this study focused on the diversity and distribution characteristics of bacteria and archaea in its sediments. The sampling points were set at four sampling points in mangrove areas along the estuaries of Luoyang River and Jinjiang River, in order to explore the influence of environmental factors on microbial diversity more comprehensively. The results show that the species richness of mangrove sediments in different areas of Quanzhou Bay is generally high and similar. Among them, the Shannon index of JJ sample is high, indicating that its species are numerous and evenly distributed, and the Faith index also shows that its phylogenetic diversity is high. However, Simpson index exceeded 0.990, indicating that some species were dominant in each sample community and the overall diversity was low. The difference in the number of OUTs in different regions may be related to the environmental conditions and human activities in each region. For example, JJ area is rich in fishery and commercial activities, and it is located at the mouth of Jinjiang River, where fresh water and seawater meet, which may affect the structure and diversity of its microbial community. LJ area is located in the mangrove area under the dam in the upper reaches of Luoyang River, which is less affected by human activities. FZ area is located at the mouth of the lower reaches of Luoyang River, and there are few human activities around it. The SS area is located in the periphery of the estuary and is greatly influenced by the marine environment. These factors may lead to the diversity and structural differences of microbial communities in different regions.

In this study, we used high-throughput sequencing technique to deeply analyze the bacterial diversity and distribution characteristics of mangrove sediments in Quanzhou Bay. The results show that Pseudomonadota is dominant at the phylum level, which is consistent with the previous research results of several mangrove ecosystems around the world. For example, studies by Nimnoi and Pongsilp (2022) in Upper Bay, Thailand, and mangrove reserves in South China and Bamenwan, China, all show that Pseudomonadota is the main dominant phylum, followed by Desulfobacterota, Bacteroidota and Chloroflexi. Ghosh et al. (2022) research on Indian mangrove ecosystem also confirmed that Pseudomonadota has the highest abundance, followed by Firmicutes, Bacteroides and Actinomycetes. These consistent results strengthen the universal dominant position of Proteus in different mangrove environments, indicating that Proteus plays an important role in the ecological function of mangrove ecosystem. Gammaproteobacteria is the most abundant taxa at the class level, which is consistent with the previous research. Gammaproteobacteria, as an important part of environmental microorganisms, includes a variety of marine bacteria and sulfide-oxidizing bacteria, which may play a key role in the decomposition of organic matter and sulfur cycle in mangrove sediments. In addition, Woeseiales is dominant at the order level, and these microorganisms are widely distributed in various environments around the world and play an important role in nitrogen cycle and carbon cycle. At the family level, Rhodobacteraceae is one of the main groups. As photosynthetic bacteria, they may play a key role in the primary production of mangrove sediments. At the genus level, Nitrosopumilus_5141 is one of the main taxa, belonging to archaea ammoxidation, which participates in ammonia oxidation reaction in nitrogen cycle, and emphasizes the importance of nitrogen cycle in mangrove ecosystem (Zheng et al., 2024). In addition, at the species level, SZUA_442_sp003235475 shows the highest abundance. Although its specific function needs to be studied, its high abundance may indicate its important role in some specific ecosystem functions. These findings not only reveal the diversity and uniqueness of microbial communities in mangrove sediments in Quanzhou Bay, but also provide important data resources, laying a foundation for further discussion on the role of microorganisms in mangrove ecosystem functions in the future. Future research should further explore the specific ecological functions of these microorganisms and their interaction with environmental factors, especially in the context of coping with climate change and human activities, how to maintain the stability and health of mangrove ecosystems will be an important research direction.

NMDS and PCA are commonly used multivariate data analysis methods, which are used to explore and visualize complex data sets, especially in microbiology and ecological research. NMDS reduces the data dimension by maintaining the relative ordering of similarity between samples, while PCA projects the data to a new orthogonal coordinate system through linear transformation to explain the variation. In this study, NMDS and PCA analysis revealed the differences of microbial communities in different mangrove sediments in Quanzhou Bay, but the results of the two methods were different, which may be related to their different ways of dealing with data variation and distance measurement. NMDS analysis shows that samples of SS, JJ and LJ gather on the left side of NMDS1 axis, which indicates that the microbial community structure in these areas is similar. However, the samples of FZ are obviously separated, indicating that the microbial community is significantly different from other areas, which may be related to its specific environmental conditions (such as soil type, nutrient level or human activities). The aggregation of samples in the same area shows that environmental homogeneity leads to the similarity of microbial community structure. PCA analysis further revealed the differences of microbial communities in different regions, and some samples gathered in NMDS but dispersed in PCA, indicating that it is necessary to comprehensively use various analytical methods to understand the microbial community structure.

The analysis of characteristic microbial species further confirms the specificity of microbial communities in different regions. In the SS region, Bacteroidota is dominant, which may be related to its high organic matter input or hydrological conditions. The JJ region has only a few characteristic species, reflecting its specific environmental pressures or niche limitations. The characteristic species in the LJ region include microorganisms related to the nitrogen cycle, such as Marinobacterium, which may be associated with the nitrogen cycling in this area (Huang et al., 2020). The FZ region has the most diverse characteristic species, including various archaea and bacteria, which may be related to its complex ecological environment and the interaction of multiple environmental factors. These results indicate that environmental factors have a significant impact on the composition and diversity of the microbial community in mangrove sediments. Microorganisms in different regions not only differ in diversity, but the types of their characteristic microorganisms also reflect their unique ecological functions and adaptation strategies. Future research could further explore the roles of these microorganisms in the ecosystem services of mangroves and their response mechanisms to environmental changes. Incorporating more environmental parameters and metabolomics data will contribute to a deeper understanding of the roles and ecological significance of microbial communities in the mangrove ecosystem.

The observed spatial patterns in microbial community structure and function have direct implications for key mangrove ecosystem services. The widespread dominance of Pseudomonadota and Chloroflexi, alongside a high predicted potential for motility and the processing of nitrogen-rich organic compounds, points to a microbial consortium highly efficient in decomposing mangrove-derived organic matter. This activity is central to nutrient cycling, particularly for nitrogen, where taxa like Nitrosopumilus and Woeseiales likely drive nitrification and nitrogen assimilation processes, potentially mitigating land-derived nitrogen loads in Quanzhou Bay. Simultaneously, this heterotrophic activity regulates the carbon sequestration potential of sediments by determining the balance between carbon mineralization and stabilization. Furthermore, microbial communities contribute indirectly to coastal protection. Through processes such as biofilm formation and the metabolic detoxification of pollutants, they enhance sediment cohesion and water quality, thereby bolstering the overall resilience of the mangrove buffer. The distinct and functionally unique community at the FZ site, correlated with specific environmental pressures, underscores that anthropogenic influences can reshape these microbially-mediated services, with implications for the long-term health and protective capacity of urban mangrove ecosystems.

The function prediction and analysis of microbial communities in sediments of different mangrove sites in Quanzhou Bay showed that all the microorganisms at the sites had the functions of Flagellarassembly, Bacterialchemotaxis, Valine, Leucine and Leucine Biosynthesis, among which Valine and Leucine and Leucine Biosynthesis had the highest proportion. Through PCA analysis of KEGG database, the contribution rate of first principal component (PC1) is 53.59%, and the contribution rate of the second principal component (PC2) is 12.79%, indicating that there are significant differences in the main pathways such as biological metabolism and signal transduction. Although there is obvious separation in PC1, the samples in the group also show significant variability, which may be related to microenvironment, sediment source and biodiversity, suggesting that even in the same mangrove area, the biological characteristics of sediments are heterogeneous and may be affected by local environmental conditions.

The results of water quality analysis in the coastal waters of Quanzhou Bay show that there are significant differences in environmental parameters in different mangrove areas. There is no significant difference in PH value between JJ and SS regions, ranging from 7.52 to 8.18, while there are significant differences in NH_4_-N and NO_3_-N among regions, among which the contents of NH_4_-N and NO_3_-N in FZ region are the highest, which are 105.50 mg/L and 422.75 mg/L respectively, while those in JJ region are the lowest, which are 62.73 mg/L and 299.00 mg/L, respectively. The value of CDC in different places is 19.34–28.15 ms/cm, and there are also significant differences. There is a significant difference in LDO between FZ and other regions, and the content ranges from 8.15 to 10.23 mg/L, but there is no significant difference among JJ, LJ and SS. Salinity showed obvious differences in all regions, and its content ranged from 15.13% to 23.00%. These results show that the water quality parameters of different mangrove areas in the coastal waters of Quanzhou Bay are affected to varying degrees, revealing the heterogeneity of environmental conditions between regions, which may be related to local pollution, biological activities or different chemical compositions of sediments.

In order to evaluate the relationship between environmental factors such as water quality and microbial community abundance, correlation heat map analysis and RDA (redundancy analysis) were carried out in this study. The results show that 30 kinds of sediment microorganisms are closely related to 6 kinds of environmental factors, among which 18 kinds of microorganisms are positively related to environmental factors and 12 kinds of microorganisms are negatively related. RDA analysis further showed that the explanation rates of community structure differences on the two sorting axes were 26.95% and 21.65%, respectively, in which microbial communities had the strongest correlation with LDO (dissolved oxygen) and the weakest correlation with NH_4_-N (ammonium nitrogen). It is particularly noteworthy that the microbial community in FZ area is positively correlated with all six environmental factors, while the samples in other areas are in the opposite trend. These results show that environmental factors have a significant impact on microbial community structure, especially LDO plays a key role in shaping microbial community structure, while NH_4_-N has a relatively small impact. The difference in response of microbial communities in different regions to environmental factors may be closely related to the unique environmental conditions and ecological characteristics of each region, which provides an important basis for further understanding the dynamics of microbial communities in mangrove ecosystems.

## 5 Conclusion

This study systematically analyzed the microbial diversity of mangrove sediments in Quanzhou Bay and its relationship with environmental factors, revealing the complexity and regional specificity of mangrove ecosystem. It is found that Pseudomonadota is the main dominant bacteria in mangrove sediments, and it is widely distributed in many mangrove areas, which is consistent with other mangrove research results in the world. In terms of community structure, NMDS and PCA analysis show that there are significant differences in microbial community structure at different points, especially the FZ area is obviously separated from other areas, which may be related to its unique environmental conditions and human activities. In addition, the microbial function prediction analysis of mangrove sediments shows that microorganisms have the functions of Flagellarassembly, Bacterialchemotaxis, valine, Leucine and isoleucine biosynthesis, and there are significant differences in metabolism and signal transduction pathways. The water quality analysis further verified that the environmental conditions in different mangrove areas in Quanzhou Bay were significantly heterogeneous, especially the distribution of environmental factors such as NH_4_-N, NO_3_-N, LDO and Salinity in different areas. These differences may affect the composition and function of microbial communities, revealing the significant regulatory effect of environmental factors on microbial community structure. RDA analysis further shows that LDO is the key environmental factor affecting microbial community structure, while NH_4_-N has little influence. In addition, the species of characteristic microorganisms in different regions also showed significant differences. For example, Bacteroidota in SS region was the most affected, while Archaea and Gammaproteobacteria were the main characteristic microorganisms in FZ region.

To sum up, the microbial community of mangrove sediments in Quanzhou Bay has high diversity and regional specificity, which is influenced by environmental factors and local ecological conditions. The results of this study not only enrich the understanding of microbial community structure and function in mangrove ecosystem, but also provide scientific basis for the protection and management of mangrove ecosystem. To build upon these findings, future research should focus on: (i) unraveling the specific plant-microbe feedbacks in the rhizosphere of dominant mangrove species (e.g., Kandelia candel) to understand vegetation-driven assembly; (ii) assessing temporal dynamics (seasonal/annual) of the community to disentangle environmental forcing from ecological succession; (iii) employing multi-omics approaches (e.g., metatranscriptomics) to move beyond functional prediction and validate the mechanistic roles of key taxa in biogeochemical cycles; and (iv) experimentally evaluating microbial community resilience under simulated climate change stressors (e.g., warming, intensified salinity) pertinent to subtropical urbanized coasts. Addressing these questions will transform our descriptive understanding into a predictive framework for managing mangrove ecosystem services under global change.

## Data Availability

The data presented in the study are deposited in the NCBI repository. Available at: https://www.ncbi.nlm.nih.gov/sra/PRJNA1167012, accession number PRJNA1167012.
